# Acceptance of New Respiratory Syncytial Virus Vaccine among Pregnant Women in Nepal for Future Routine Immunization: A Descriptive Cross-sectional Study

**DOI:** 10.31729/jnma.8622

**Published:** 2024-06-30

**Authors:** Santosh Adhikari, Ram Hari Chapagain, Jessica Maharjan, Kshitij Kunwar, Sudip Pudasaini, Pramod Singh, Abhiyan Gautam, Tribhuwan Bhattarai, Srijana Bhattarai

**Affiliations:** 1 Kanti Children's Hospital, Maharajqunj, Kathmandu, Nepal; 2 National Academy of Medical Sciences, Kathmandu, Nepal; 3 Shankarapur Hospital, Kathmandu, Nepal; 4 Kakani Primary Health Care Center, Nuwakot, Nepal; 5 Family Welfare Division, Teku, Kathmandu, Nepal; 6 Paropakar Maternity Hospital, Kathmandu, Nepal; 7 Research and Innovation Foundation Nepal, Kathmandu, Nepal

**Keywords:** *antenatal vaccination*, *Nepal*, *respiratory syncytial virus*, *RSV vaccination*, *vaccine acceptance*

## Abstract

**Introduction:**

Children are at greatest risk for severe illness from Respiratory Syncytial Virus (RSV). The aim of the study was to find out the knowledge of RSV, practice and knowledge about vaccination during pregnancy and the willingness to accept vaccines against RSV during pregnancy in the future among mothers needs to be understood which would add up information for stakeholder and policy makers.

**Methods:**

This is a descriptive cross-sectional study. A preformed Performa was used for face-to-face interview was conducted among 340 pregnant women who visited the Antenatal clinic from 15 Oct 2023 to 30 Nov 2023 in their second and third trimester. Socio-demographic characteristics, knowledge and the attitude concerning antenatal vaccination affecting the acceptance of RSV vaccine were evaluated from the interview.

**Results:**

The mean age was 28.4 years, with 310 (91.18%) already having at least one child. Six (1.76%) participants had previously heard about RSV, and 325 (95.59%) were aware of the problem caused by RSV after they were briefly explained about it in their local language. A total of 246 (72.35%) of the mothers expressed willingness to be vaccinated themselves rather than vaccinating their children if such an option existed. Only 2 (0.59%) participants were familiar with nasal vaccines, and only 18 (5.29%) believed in such vaccines being effective. Despite this, almost all participants 339 (99.71%) in the study demonstrated willingness to receive additional antenatal vaccines if approved for use in future.

**Conclusions:**

The study showed a limited understanding of RSV in children among pregnant women in Nepal. However, they are aware of the impact of bronchiolitis and expressed a strong willingness to undergo maternal vaccination against RSV.

## INTRODUCTION

Respiratory diseases most common in children and mostly are due to respiratory syncytial virus with high indices of hospital admission in children under 5 years of age.^1,2^ At present, there are multiple approaches to safeguarding children from RSV, including maternal immunization, monoclonal antibody treatment and childhood vaccination.^3^ The approval for maternal vaccination for RSV has been started in different countries in Europe so that the global burden of RSV can be tackled as well as many childhood vaccines in pipeline.^4-6^

The successful implementation of any maternal vaccine program depends on women's willingness to accept vaccination. Therefore, our objective was to explore maternal acceptance during pregnancy for a new vaccine designed to safeguard both mothers and their children. We also aimed to gauge their willingness to adopt the new vaccine should it receive government approval. This information is one of the crucial for stakeholders in planning and formulating policies for future vaccines once they are approved by government for the future vaccine administered during pregnancy.

## METHODS

This is a descriptive cross-sectional study to gather quantitative data from study participants. This study was conducted in the Antenatal Clinic of Paropakar Maternity Hospital, (PMH), Kathmandu, which is the largest tertiary care center and a government maternity hospital in Nepal. PMH has a substantial capacity to accommodate a large number of pregnant women. The hospital's accessibility to diverse patient groups makes it representative of the Nepali population across various categories.

The study included pregnant women in their second and third trimesters who attended antenatal checkups at the Antenatal Clinic of Paropakar Maternity Hospital and provided consent to participate in the study. A validated structured local language questionnaire, with questions related to demographic background, knowledge and the attitude concerning antenatal vaccination, was used for data collection. The responses were collected and entered using Microsoft Excel 2019 which was analyzed using R version 4.3.1.

The data collection was carried out from 15 Oct 2023 to 30 Nov 2023. The tabulated value of Z at 95% level of significance is 1.96, Z^2^ = (1.96)^2^ = 3.84; prevalence(p) = 7%^7^; q = 100-p = 83; e^2^ = 25. The calculated sample size was calculated to be 320. A convenience sampling method was used for the study participants' enrollment. An additional 21 (6.56%) participants (a total of 341 participants) were approached considering the possible non-response from the approached participants.

The Nepalese participant who had visited the antenatal clinic of PMH during her second or third trimester of pregnancy and provided consent to take part in the study were included in the study. Those participants who were not Nepalese women, who were not sure about her gestation age and who did not provide consent to participate in the study were excluded in the study. This study excluded women in their first trimester of pregnancy, as the vaccine against RSV is intended for pregnant women in their third trimester. This study was conducted after ethical clearance from IRC of Paropakar Maternity Hospital (PMH), Thapathali, Kathmandu (ref. no. 64/300 for protocol registration no. 4064/2080). Microsoft Excel 2019 was used to encode the data and R version 4.3.1 to analyze data. Data was summarized by calculating descriptive statistics, including frequencies and percentages. Univariate descriptive statistics were performed for each variable, including measures of frequency and central tendency.

## RESULTS

Among 340 participants, 231 (67.94%) of the study participants were aged between 25 to 30 years with the mean age being 28.4 years. Out of 231 patients, 214 (62.94%) had already have one pregnancy before. (Table 1).

**Table 1:** Background of study participants (n= 340).

SN	Variables	Response	n (%)
1	Participant's age (in number of years completed)	20 to 24	64 (18.82)
		25 to 30	231 (67.94)
		31 to 35	43 (12.64)
		36 to 40	2 (0.59)
3	Gestational age of pregnancy (in weeks)	20 - 28	145 (42.65)
		>28	195 (57.35)
4	Pregnancy count (including current one)	1 (Primigravida)	29 (8.53)
		2(Second gravida)	214 (62.94)
		3 (Multigravida)	97 (28.53)
5	Ethnic group	Brahmin / Kshetri	140 (41.18)
		Janajati	162 (47.65)
		Dalit	16 (4.71)
		Madhesi	22 (6.47)
6	Current number of children	None	30 (8.82)
		1	211 (62.06)
		2	98 (28.82)
		3 or more	1 (0.29)

There were only 6 (1.76%) participants responding that they had heard about RSV and 334 (98.24%) participants responded that they had never heard about RSV. Out of 6 participants who had knowledge of RSV, 3 (50%) knew someone who had been infected with RSV and 1 (16.67%) participant's child have been infected by RSV (Table 2).

**Table 2:** Knowledge and perception of study participants on Respiratory Syncytial Virus (n = 340).

SN	Variable	Response	Number (%) of response
1	Awareness of Respiratory Syncytial Virus (RSV) in children	No, I have not heard anything about it.	334 (98.24)
		Yes, I have heard about it but do not know it in detail	2 (0.59)
		I know something about it	3 (0.89)
		I have heard about it and have good knowledge about it	1 (0.29)
2	Experience with RSV (n = 6)	I don't have any experience	3 (50.00)
		I know someone who has been infected by it	3 (50.00)
3	History of RSV infection in one’s child (n = 6)	Present	1 (16.67)
		Absent	3 (50.00)
		I don't have a child yet (Not applicable)	2 (33.33)
4	RSV infection in children risk perception (n = 6)	There is possibility of infection	4 (66. 67)
		There is no possibility of infection	0 (0.00)
		I don't know	2 (33.33)
5	RSV infection in children severity perception (n = 6)	No problem is seen	0 (0.00)
		Non-severe problems might be seen	3 (50.00)
		Severe problems might be seen	1 (16.67)
		I don't know	2 (33.33)
6	Awareness of Bronchiolitis in children	I have never heard about it	15 (4.41)
		I have heard about it but don't know about it	283 (83.24)
		I know a little about it	5 (1.47)
		I know something about it and about other people who have experienced it	32 (9.41)
		I know about it and I have experienced this	5 (1.47)
7	Bronchiolitis in children risk perception	There is possibility of infection	320 (94.12)
		There is no possibility of infection	1 (0.29)
		I don't know	19 (5.59)
8	Bronchiolitis in children severity perception	No problem might be seen	11 (3.34)
		Non-severe problems might be seen	208 (61.18)
		Severe problems might be seen	101 (29.71)
		I don't know	20 (5.88)

Most participants 339 (99.41%) were willing to accept an additional three or more vaccines during pregnancy, while 123 (36.18%) of them were willing to accept 5 or more additional vaccines during pregnancy.

**Table 3:** Study participants’ willingness to use vaccines for Respiratory Syncytial Virus.

SN.	Variables	Response	Number (%) of response
1	Awareness regarding Nasal vaccines	Present	2 (0.59)
		Absent	338 (99.41)
2	Belief in Nasal vaccines’ effectiveness	Believes	18 (5.29)
		Doesn't believe	322 (94.71)
3	Preferred recipient for RSV vaccine	I'd vaccination myself (vaccine made for mother)	246 (72.35)
		I'd vaccination my child (vaccine made for child)	94 (27.65)
4	Willingness to use RSV vaccine (if included in the national immunization schedule)	I'd definitely use it	142 (41.76)
		I'd probably use it	5 (1.47)
		I'd possibly use it	192 (56.47)
		I'd probably not use it	1 (0.29)
5	Additional number of vaccine acceptance during pregnancy	0	0 (0.00)
		1	1 (0.29)
		2	1 (0.29)
		3	212 (62.35)
		4	3 (0.88)
		5 or more	123 (36.18)

## DISCUSSION

Vaccines have consistently been regarded as a safe and efficient method for preventing numerous infectious diseases.^8^ Maternal vaccination is an effective means of protecting pregnant women, their fetuses, and infants from vaccine-preventable infections.^9^ Despite the availability of sufficient safety data to support the use of vaccines during pregnancy, maternal immunization remains an underutilized method of disease prevention, often because of concerns from both healthcare providers and pregnant women about vaccine safety.^9^ In Nepal, Tetanus Diphtheria (Td) is the sole vaccine administered to expecting mothers as per the National Immunization Schedule of Nepal.^10^ Through this study, we have tried to explore the acceptance of potential new vaccines among pregnant women. The antibody produced by effective vaccination will transfer through the placenta and will protect the newborn.^11^ Most mothers (72.35%) said that they would rather vaccinate themselves instead of their child if doing so would also provide the benefit to the child. Almost all of the participants (97.71%) said that they were willing to take the vaccine for RSV if it is included in the national immunization schedule. Most participants were willing to accept up to 3 additional vaccines during pregnancy while (36.18%) of them were ready to accept 5 or more additional vaccines during pregnancy. Our study showed that most of the participants were below 30 years, showing young mothers mostly in the third trimester having previous children. There were mothers who already had one child with them. The important finding was the maternal acceptance of RSV vaccination during pregnancy for the safety of one's child. Nasal vaccines are very easy to administer in children as it does not need injection and there are less chances of adverse events though there are scheduling challenges in the national immunization program.^12-14^ In our study, only two participants were aware of any kind of nasal vaccine and around 5.29% of study participants believed that a nasal vaccine would be effective this and other disease.

In our study, there were a negligible number of participants who were aware about RSV which contrasts with the findings from the study done in England and Australia, where there were 29% and 17% participants who were aware about the virus.^15,16,^ This difference may be attributed to greater awareness in those regions compared to our part of the world. Limited number of research papers in Low middle income countries and in neighboring countries has hindered us from observing the trend of awareness of RSV among people.^17^ Despite an increased incidence of RSV globally, public awareness and understanding of this disease remains limited. Furthermore, there is no definite correlation between RSV and temperature variation.^14^ This has created a problem for effective control and prevention of this disease.

Some respondents mentioned being familiar with respiratory illnesses like pneumonia and symptoms such as noisy breathing, which they perceived as less severe but they were unaware about the term 'RSV' specifically. Similar findings were observed in a study done in Kenya where participants were asked about RSV, but none of them seemed to be aware about it, but when explained about the problems caused by it in their local language, there was an increase in the number of responses indicating awareness to the problems associated with it.^15,18^ This signifies that awareness should be raised to make people understand about it. To address this issue, awareness should be raised among the general population and among healthcare workers as well, as their knowledge can help in reducing the mortality caused by RSV.

The acceptance of vaccines is higher in our part of the world, at almost 97%, which is much higher than the western world where there was lower acceptance of recommended vaccines.^19^ This vaccine acceptance for future RSV vaccine was quite different in our study than the study done in developed world with less than 30% acceptance rate was seen for RSV vaccination.^20^ In our study, almost three-fourths of the participants showed interest in receiving RSV vaccination. This could be attributed to the belief in effectiveness of vaccines in preventing disease in childhood, given the widespread coverage of TT vaccine in pregnancy, which has caused a significant decrease in the number of cases of neonatal tetanus.^21^

This study highlights the need for generating awareness about RSV and RSV vaccination. This can be done by educating the health workers around the globe, who can disseminate this information to the general population. Additionally, this study reveals that mothers are more receptive to antenatal vaccines for the well-being of their child, as opposed to vaccinating their child directly. This information is valuable to stakeholders aiming to address RSV-related issues, and designing strategies to promote future vaccination against RSV.

There are a few limitations to our study, including the absence of a qualitative component where the knowledge and the thinking of the mothers for the disease burden and the sequel of the disease could have been taken, which could have provided a more comprehensive understanding of the issues. Also, as the study was confined to a single center, the generalizability of the findings can be limited. Future studies can be carried out to address these limitations and contribute to filling the gaps identified in the study as well as the convenience sampling technique.

## CONCLUSIONS

The understanding of RSV in children among pregnant women in Nepal is very limited however the awareness of impact of bronchiolitis is more which is similar to findings of other countries. The participants showed the strong willingness to participate in maternal vaccination once it is available and recommended by government. There was less confidence in nasal vaccination for children against RSV. This highlights the need for education on RSV for pregnant women and suggests that maternal vaccination might be a preferred choice, possibly influenced by concerns or preferences related to vaccination methods. The awareness regarding RSV and effectiveness of vaccines, especially those given through nasal routes, should be made.

Recognizing and addressing these factors is important for developing effective vaccination strategies to safeguard both pregnant women and their infants from RSV-related complications, to minimize the mortality and morbidity from RSV and to promote effective strategies in reducing the burden of RSV in children in Nepal.
